# The modified Dunn procedure for slipped capital femoral epiphysis does not reduce the length of the femoral neck

**DOI:** 10.12669/pjms.322.8638

**Published:** 2016

**Authors:** Dan Cosma, Dana Elena Vasilescu, Andrei Corbu, Mădălina Văleanu, Dan Vasilescu

**Affiliations:** 1 Dan Cosma, MD, MSci, PhD. Department of Pediatric Orthopedics, University of Medicine and Pharmacy “Iuliu Haţieganu”, Cluj-Napoca, Romania; 2 Prof. Dana Elena Vasilescu, MD, PhD. Department of Pediatric Orthopedics, University of Medicine and Pharmacy “Iuliu Haţieganu”, Cluj-Napoca, Romania; 3 Andrei Corbu, MD. Department of Pediatric Orthopedics, University of Medicine and Pharmacy “Iuliu Haţieganu”, Cluj-Napoca, Romania; 4 MădălinaVăleanu, PhD. Department of Medical Informatics and Biostatistics, University of Medicine and Pharmacy “Iuliu Haţieganu”, Cluj-Napoca, Romania; 5 Dan Vasilescu, MD. Department of Radiology, University of Medicine and Pharmacy “Iuliu Haţieganu”, Cluj-Napoca, Romania

**Keywords:** Slipped capital femoral epiphysis, Modified Dunn procedure, Pinningin situ, Open surgical dislocation, Femoroacetabularimpingement (FAI)

## Abstract

**Objective:**

The treatment of slipped capital femoral epiphysis (SCFE) is evolving, with the development of new surgical techniques. |We wanted to study if modified Dunn procedure restores the normal alignment of the proximal femur and the risk of avascular necrosis is increased.

**Methods:**

This is a single centre, retrospective study, comparing the outcomes of in situ pinning and modified Dunn procedure. Between 2001 and 2014, 7 children (7 hips) underwent the modified Dunn procedure and 10 children (10 hips) pinning in situ for stable and unstable SCFE. Mean age of the patients was 12.7 years with a median follow-up of 18 months.

**Results:**

The radiological parameters improved in the modified Dunn procedure group, while the length of the femoral neck didn’t change significantly (p=0.09). Postoperative clinical outcomes were slightly better in the modified Dunn procedure group (6 hips out of 7 had good and excellent results) compared to the pinning in situ group (8 good and excellent results out of 10 hips) (p=0.04). No avascular necrosis was found and there were no cases of chondrolysis.

**Conclusion:**

Radiographic parameters of the proximal femur assessed in our study improved in all hips that underwent modified Dunn procedure, without the creation of secondary deformities.

## INTRODUCTION

Slipped Capital Femoral Epiphysis (SCFE) is the gradually acquired mal-alignment of the proximal femoral metaphysis and capital epiphysis. Weakening of the proximal femoral physis may lead to posterior slip of the capital epiphysis relative to the femoral neck.

In situ fixation of SCFE has a low surgical risk and the remodeling potential has been advocated for restoration of the disturbed anatomic axes. Recently, this concept has been criticized with the recognition that the head-neck offset will remain abnormal leading to potential femoroacetabular impingement (FAI). Even mild slips will create a cam configuration of the anterior head-neck junction, leading to early acetabular damage.^1^

In this study we investigated the outcomes of children with SCFE treated with the modified Dunn procedure in a centre without previous experience in surgical hip dislocation, reporting the results against another group of children treated with pinning in situ procedure in the same centre.

## METHODS

From December 2011 to December 2014, we conducted a monocentric, retrospective, continuous study, evaluating the clinical and radiological evolution of patients presenting with SCFE, treated in our service. The inclusion criteria in the present study were a diagnosis of SCFE, without other congenital or acquired hip deformity, deformity not treated previously in other hospital and a minimum of one year follow-up after the initial surgery. The exclusion criteria of the study were: patients with other congenital or acquired hip deformity, patients treated previously in other hospitals and those with less than one year of follow-up.

We obtained informed consent of the parents of patients prior to being included into the study and the research was approved by the local ethical committee. The study was performed in accordance with the Ethical standards of the 1964 Declaration of Helsinki.

Hospital records, operative records, outpatient records, and follow-up radiographs of all eligible subjects were retrospectively reviewed to determine the demographic information, duration of symptoms, type of fixation, slip angle, presence of osteonecrosis, and any additional complications. Medical records were assessed by one of the authors not involved in the clinical care of the patients.

Patients were divided into acute and chronic cases of slipped capital femoral epiphysis by reviewing their initial medical records: acute if the duration of symptoms was less than three weeks, and chronic if more; and if the prodromal symptoms (vague groin pain, upper or lower thigh pain, limp)were present prior to slip, it was considered as acute on chronic.^2^ The slips were described as stable or unstable as suggested by Loder.^3^

On frog lateral view, the preoperative Southwick angle^4^ was measured and the contour of the femoral head-neck junction was assessed by measurement of the head-neck offset.^5^ The severity of the displacement was subdivided into three stages: mild (slip angle inferior to 300), moderate (slip angle between 300 and 600), or severe (angle more than 600).^4^ On a AP view of the pelvis with the limbs in the neutral position, the length of the femoral neck on the affected and on the unaffected sides were measured, based on the method described by Bleck.^6^ The radiological measurements were done using the picture archival and communication system (PACS) (OFFIS e.V., R&D Division Health, 2013, Oldenburg, Germany). All radiographs were assessed by one author (DV).

All patients presented to our hospital were treated surgically. One surgeon (DV) performed only in situ pinning, while another surgeon who specialized in these procedures (DC) performed the modified Dunn procedure. No preoperative traction or closed reduction of the SCFE was tried out in the hospital.

Treatment results were assessed using the Heyman and Herndorn classification system.^7^ A hip is considered excellent if it has normal ROM, and there is no limp and no pain; good if there is no limp, no pain, and slight limitation of internal rotation but external rotation beyond neutral; fair if there is no limp, no pain, and slight limitation of abduction and internal rotation; or poor if there is a mild limp, slight pain after strenuous exercise, and slight limitation of internal rotation, abduction, and flexion. A hip is considered failed if there is a limp, pain on activity, and marked limitation of motion requiring reconstructive surgery or progressive radiographic changes are seen.^8^

Postoperative radiographic parameters were also assessed similar to the preoperative workout. Radiological evidence of the avascular necrosis of the femoral head (AVN) was recorded at the latest follow-up. Chondrolysis was diagnosed if there was loss of 50% of the joint space.^9^

The modified Dunn procedure was performed by one of the surgeon (DC) according to a previously described technique.^10^ The patient was placed in a full lateral position and the transtrochanteric surgical dislocation of the hip performed.^11^ The femoral head was then dislocated from the acetabulum and the ligamentum teres was transected. The epiphyseal perfusion was checked by drilling a 2-mm hole in the anterior femoral head.^12^ The femoral head was reduced back and the soft tissue retinaculum was dissected after the proximal portion of the stable trochanter was carefully removed.^13^ The femoral neck was completely exposed after periosteal dissection. Using a curved chisel, the epiphysis was gradually separated from the metaphysis and the posterior callus was trimmed from the metaphysis. The femoral head was then reduced back to the femoral neck in an anatomic alignment and fixed with two 6.5-mm cannulated screws. The greater trochanter was refixed with two 3.5-mm screws. Postoperatively, patients started the physical therapy for 6 weeks non-weight bearing, followed by more 6 weeks weight bearing with crutches.

In situ pinning was performed by one surgeon (DV) with the patient lying supine on a normal radiolucent table. A guide wire was introduced in the anterolateral aspect of the femoral neck aiming towards the center of the femoral head in the AP and lateral views. One 6.5-mm cannulated screw was then placed through a small anterolateral skin incision. Patients were allowed partial weight bearing with crutches for 6 weeks.

The statistical analysis was performed using the SPSS 15.0 software (SPSS Inc., Chicago, USA). We tested normal distribution using the Kolomogorov – Smirnov test. Descriptive statistics were performed on all variables of interest. The mean and SD were reported for continuous variables when normally distributed and the median and interquartile range when not normally distributed. The group differences in the pre- and postoperative radiographic normal distributed variables were compared using Student t-test. Non-parametric Mann – Whitney and Kruskall – Wallis tests were used for non-parametric variables. We used Chi-Square or Fisher Exact tests for comparison between qualitative variables. Pearson correlation coefficient (for normal distributed variables) or Spearman correlation coefficient (for non-Gaussian distribution) were calculated. *P* values are reported throughout and are considered significant when <0.05.

## RESULTS

A total of 17 patients were identified, among which 12 were boys and 5 were girls, with a mean age of 12.7 years (range 11 – 14 years).

Based on Southwicks`s classification, we identified 11 hips with a chronic slip, 3 with acute slip, and 3 with acute on chronic slip. Among 17 patients, 6 underwent in situ pinning for stable slips and 4 children underwent the same for unstable slips. Six patients underwent safe surgical dislocation and modified Dunn procedure for stable slips and one child underwent the same for unstable slip.

Seven patients underwent the modified Dunn procedure (between 2011 and 2014) and 10 had in situ pinning (between 2011 and 2014). The two groups were statistically comparable regarding sex, age at surgery, affected side, duration of symptoms, chronicity, severity, and follow-up period (Table-I).

**Table-I:** Descriptive statistics by treatment group.

Variable	Modified Dunn procedure group	Pinning in-situ group	p-value
*Sex*			
Female – number (%)	-	7 (70%)	
Male – number (%)	7 (100%)	3 (30%)	0.23
*Age at surgery (yrs)*			
Median (25%– 75%)	13 (12 – 13)	13 (12 – 14)	0.96
*Affected side*			
Left – number (%)	4 (57.1%)	5 (50%)	
Right – number (%)	3 (42.9%)	5 (50%)	0.99
*Duration of symptoms (wks)*			
Median (25%– 75%)	5 (3.5 – 5.5)	5 (3 – 7)	0.74
*Chronicity*			
Acute – number (%)	2 (28.6%)	2 (20%)	
Chronic – number (%)	4 (57.1%)	6 (60%)	
Acute on chronic – number (%)	1 (14.3%)	2 (20%)	0.9
*Stability*			
Stable – number (%)	6 (85.7%)	6 (60%)	
Unstable – number (%)	1 (14.3)	4 (40%)	0.34
*Follow-up (months)*			
Median (25%– 75%)	12 (8.5 – 23)	22 (15 – 29)	0.36

The median follow-up between the time of the surgical intervention and the latest follow-up (December 2014) was 18 months (range 6 months – 35 months).

**Fig.1:**
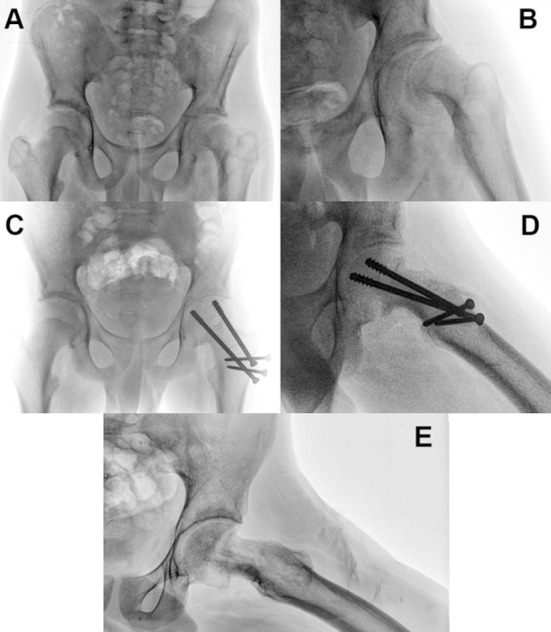
This is an illustrative case of a 13-year-old boy with a stable left slipped capital femoral epiphysis. (A) Preoperative anteroposterior and (B) lateral views. (C – D) Early postoperative anteroposterior and lateral views after modified Dunn procedure. (E) 18 months after the modified Dunn procedure and screws removal. The physis and trochanteric osteotomy healed without complications. There is no evidence of AVN or chondrolysis. The anterior head-neck offset is fully restored.

Improvement of the radiographic parameters (Table-II) in the modified Dunn procedure group (Fig.1) didn’t result in the shortening of the femoral neck (mean neck length on the operated side=99.1mm compared to mean neck length on the normal side=101mm, p=0.09).

**Table-II:** Pre- and postoperative radiographic parameters

*The modified Dunn procedure group*			
*Variable*	*Preoperative*	*Postoperative*	*p value*
Southwick angle (0)	68 (64 – 71.5)	9 (7.5 – 13.5)	<0.001
Head – neck offset (mm)	-4 (-5.25 – -3.65)	4.2 (3.2 – 5.3)	<0.001
Neck length (mm)	101 (100.2 – 102.7)	99.1 (98.15 – 100.25)	0.09
*The pinning in situ group*			
*Variable*	*Preoperative*	*Postoperative*	*p value*
Southwick angle (0)	34 (23 – 46)	32.5 (21 – 44)	0.02
Head – neck offset (mm)	-4.7 (-5.7 – -3.4)	-4.3 (-5.2 – -2.9)	0.04
Neck length (mm)	99.95 (98.5 – 102)	99.6 (98.8 – 100.9)	0.06
*Postoperative radiographic parameters by procedure*			
*Variable*	*Dunn*	*Pinning in situ*	*p value*
Southwick angle (0)	9 (7.5 – 13.5)	32.5 (21 – 44)	<0.001
Head – neck offset (mm)	4.2 (3.2 – 5.3)	-4.3 (-5.2 – -2.9)	<0.001
Neck length (mm)	99.1 (98.15 – 100.25)	99.6 (98.8 – 100.9)	0.69

In the in situ pinning group, the head-neck offset remained negative while the Southwick angle slightly improved (Table-II).

At latest follow-up the mean Southwick angle was lower in the modified Dunn procedure group compared with the in situ pinning group. The mean head-neck offset also was better in the modified Dunn group (Table-II).

Postoperative clinical outcomes, assessed by one of the authors (AC), were slightly better in the modified Dunn procedure group (6 hips out of 7 had good and excellent results according to the Heyman and Herndorn classification) compared to the pinning in situ group (8 good and excellent results out of 10 hips) (p=0.04). Within our series the positive impingement test (1 hip in the modified Dunn procedure group and 2 hips in the in situ pinning group) at the latest follow-up was not correlated with the type of surgical procedure (p=0.48).

**Fig.2:**
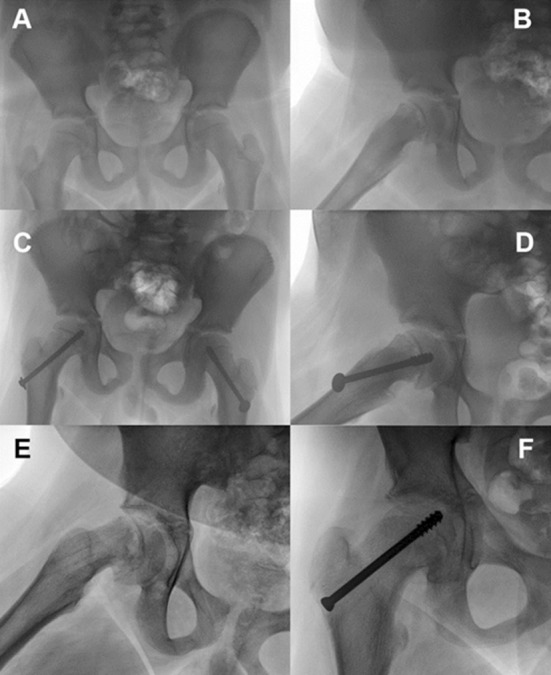
This is an illustrative case of an 11-year-old boy with a stable right SCFE. (A – B)Anteroposterior and lateral views before pinning in situ.(C – D) Postoperative anteroposterior and lateral views after pinning in situ. (E – F) Anteroposterior and lateral views after screws removal and re-slip of the right capital femoral epiphysis after 18 months.(G) Lateral view after re-pinning in situ.

With the numbers available, the rate and severity of the complications in both two groups were low. No avascular necrosis was found and there were no cases of chondrolysis. In the modified Dunn group, two patients underwent two subsequent surgical procedures: removal of the lateral protruding screws. In the in situ pinning group, three patients underwent removal of a lateral protruding screws and one patient underwent re-pinning of the re-slip after screw removal (Fig.2).

## DISCUSSION

The treatment of slipped capital femoral epiphysis is evolving and a consensus guideline is expected. Several surgical procedures have been used to stabilize and produce closure of the epiphyseal plate, and no single method has gained universal acceptance.^14^

During the last two decades, the recognition that even small slips may produce cam deformity of the proximal femur, responsible for frequently observed acetabular cartilage damage, gave rise to doubts about this treatment options.^15^–^17^

The present study was conducted to answer the following questions: (1) Is the modified Dunn procedure, performed by a trained surgeon, feasible and repeatable in an independent centre; if patients with SCFE treated by modified Dunn procedure during the learning curve showed (2) improved radiographic parameters, without (3) secondary shortening of the femoral neck.

Radiographic parameters of the proximal femur assessed in our study improved in all hips that underwent modified Dunn procedure. Novais et al. compared the outcomes of the 15 SCFEs treated by modified Dunn procedure to those of 15 hips pinned in situ. They reported the improvement of all radiographic parameters in the modified Dunn procedure group, with fewer unplanned reoperations in their patients.^8^ Arora et al.^18^ also reported better outcomes in the modified Dunn procedure compared to the pinning in situ group (eight children underwent capital realignment and eighteen children underwent pinning in situ procedure).

Unlike the other cuneiform osteotomies^19^,^20^, which require substantial femoral neck shortening to ensure tension-free correction of the femoral epiphysis, this modified Dunn approach allows safe reduction by removal of the posterior callus and thinning of the femoral neck and hence should minimize leg length differences. In our series, we didn’t notice significant shortening of the femoral neck on the operated side, comparing to the unoperated side.

Our data suggest capital realignment of SCFE with open physes through the surgical dislocation approach can be performed with low complication rates. We believe the technique is most appropriate for moderate to severe SCFE and especially for unstable SCFE. The safe execution of this procedure requires full understanding of the vascular anatomy of the hip by the surgeon. This procedure restores the proximal femoral anatomy and although we do not have long-term results, we assume restoration of normal anatomy would lead to good long-term outcomes. This procedure is technically demanding; however, we believe it is worth the investment of effort and skill for a condition that could have lifelong consequences in an otherwise young patient. In situ pinning procedure was used to stop further slip progression and osteonecrosis in stable and unstable slips. According to our results, even after fixation, the hip remodeled into an abnormal femoral head-neck junction, with the potential for future development of femoroacetabular impingement. On the other hand, the rate of osteonecrosis was null following this type of treatment.

In our series, the modified Dunn procedures were performed for severe slips. Mild slips can be treated with pinning in situ, but severe slips require anatomical restoration of the proximal femur configuration. Novais et al.^8^ and Arora et al.^18^ also performed the capital realignment procedure on severe slips. Newer approaches recommend definitive capital realignments procedures also on mild slips.^1^ Our osteonecrosis rate was null both in the pinning in situ group and modified Dunn procedure group. For this reason, we could not state that the rate of osteonecrosis is influenced by the surgical procedure, as Alshryda et al. reported.^19^ Novais et al.^8^ reported one case of osteonecrosis in the pinning in situ group and one case in the modified Dunn procedure group. Slongo et al. also reported one case of osteonecrosis in nine SCFE cases treated by subcapital realignment procedure.^21^ On the other hand, Masse et al.^22^ and Ziebarth et al.^23^ reported no cases of osteonecrosis following modified Dunn procedure. Novais et al.^8^ and Souder et al.^24^ concluded that there was no clear relationship between osteonecrosis rate and treatment method.

### Limitations of the study

Firstly, it was a small series of 17 consecutive patients and 17 affected hips. The potential selection bias was minimized by including all patients from our database that met the inclusion criteria. Secondly, the follow-up period was quite short (median 18 months) for classical hip surgery, which limit the general conclusions. The inter observer variability was eliminated because only one of the authors performed the radiographic evaluations, and all modified Dunn procedures were performed by the same surgeon, using the same standard protocol.

Although, the presented cases represented our learning curve, the postoperative results were consistent with the data in the literature and the radiological and functional improvements are evident.

Our results support the idea of restoring the normal anatomy of the proximal femur in SCFEs with a very precise and rigorous technique through the modified Dunn procedure. This, is a powerful tool for correcting the femoral head-neck junction deformities, with the potential to eliminate the anatomical anomalies that has the potential to develop into the femoroacetabulat impingement. The outcomes in an independent centre, without prior experience in surgical hip dislocation, are comparable to those obtained in the traditional hip preservation surgery centers, after previous surgical training and strictly respecting the procedures.
